# Studying the Polarization Switching in Polycrystalline BiFeO_3_ Films by 2D Piezoresponse Force Microscopy

**DOI:** 10.1038/srep12237

**Published:** 2015-07-20

**Authors:** Yaming Jin, Xiaomei Lu, Junting Zhang, Yi Kan, Huifeng Bo, Fengzhen Huang, Tingting Xu, Yingchao Du, Shuyu Xiao, Jinsong Zhu

**Affiliations:** 1 National Laboratory of Solid State Microstructures and Physics School, Nanjing University, Nanjing 210093, P. R. China; 2 Department of Physics, China University of Mining and Technology, Xuzhou 221116, People’s Republic of China; 3 College of Science, Hebei United University, Tangshan 063009, P. R. China; 4 Collaborative Innovation Center of Advanced Microstructures, Nanjing University, Nanjing 210093, P. R. China

## Abstract

For rhombohedral multiferroelectrics, non-180° ferroelectric domain switching may induce ferroelastic and/or (anti-)ferromagnetic effect. So the determination and control of ferroelectric domain switching angles is crucial for nonvolatile information storage and exchange-coupled magnetoelectric devices. We try to study the intrinsic characters of polarization switching in BiFeO_3_ by introducing a special data processing method to determine the switching angle from 2D PFM (Piezoresponse Force Microscopy) images of randomly oriented samples. The response surface of BiFeO_3_ is first plotted using the piezoelectric tensor got from first principles calculations. Then from the normalized 2D PFM signals before and after switching, the switching angles of randomly oriented BiFeO_3_ grains can be determined through numerical calculations. In the polycrystalline BiFeO_3_ films, up to 34% of all switched area is that with original out-of-plane (OP) polarization parallel to the poling field. 71° polarization switching is more favorable, with the area percentages of 71°, 109° and 180° domain switching being about 42%, 29% and 29%, respectively. Our analysis further reveals that IP stress and charge migration have comparable effect on switching, and they are sensitive to the geometric arrangements. This work helps exploring a route to control polarization switching in BiFeO_3_, so as to realize desirable magnetoelectric coupling.

It is known that, non-180° ferroelectric domain switching is significant in rhombohedral ferroelectrics. 180° domain switching doesn’t change the crystal lattice structure of rhombohedral materials, while non-180° ferroelectric domain switching may change the elastic condition and magnetic order parameters. On the one hand, elastic interactions induced by non-180° domain switching tend to destabilize switched domains, and result in the disappearance of non-volatile information storage^1^,^2^,^3^; On the other hand, non-180° domain switching under electric field can realize magnetoelectric coupling in rhombohedral ferroelectrics^4^,^5^,^6^,^7^,^8^,^9^. Therefore, the understanding of the intrinsic switching characterization is crucial for further manipulation and application of rhombohedral ferroelectrics.

BiFeO_3_ (BFO), the only known single-phase multiferroic material at room temperature till now^7^,^9^,^10^,^11^,^12^, has attracted remarkable attention because of its potential applications in eco-friendly multistate memory devices on the basis of electric and magnetic mutual modulation^13^,^14^,^15^, of which the cornerstone is the polarization switching under electric field. BFO has a rhombohedrally distortion (R3c space group) perovskite structure^16^ with rotation of oxygen octahedral and polarization distortion along the pseudocubic [111] direction^17^. Correspondingly, it possesses eight possible polarization directions and three switching angles (71°, 109° and 180°)^12^. A lot of effort has been put into understanding the switching kinetics of BFO ferroelectric domains. Kalinin *et al.*^18^ observed the polarization switching using Scanning Surface Potential Microscopy. Subsequently, Transmission Electron Microscopy^19^,^20^,^21^ and Piezoresponse Force Microscopy (PFM)^1^,^22^,^23^,^24^,^25^,^26^,^27^,^28^,^29^,^30^,^31^,^32^,^33^,^34^,^35^,^36^ were employed for ferroelectric domain characterization and investigation.

For epitaxial BFO films of known orientation, the direction of polarization can be easily found out through the analysis of piezoresponse signals that are along out-of-plane (OP) and one in-plane (IP) directions, and the switching angles^8^,^24^ could be obtained by comparing the polarizations before and after poling. Utilizing PFM characterization of epitaxial BFO films, Zavaliche *et al.* studied the priorities of polarization switching with different angles under various poling voltages^24^, and Baek *et al.* investigated the relaxation route of switched domains^1^.

Compared with epitaxial films, grains and domains in polycrystalline samples are less constrained. Therefore, the study on polycrystalline BFO films may provide more information that helps to unveil the intrinsic characterization of polarization switching. However, there are few reports focusing on polycrystalline BFO samples, possibly because of the difficulty in identifying the polarization vector^31^,^32^,^37^,^38^. A three-dimensional (3D) mapping method, in which piezoresponse signals from one OP and two IP directions were obtained through sample-rotating^29^,^39^,^40^,^41^, was proposed and proved to be effective for some ferroelectric materials.

In this paper, we manage to determine the angles of polarization switching in polycrystalline BFO films from 2D PFM^41^,^42^ signals (along one OP and one IP directions). Similar to that reported for epitaxial films^24^, polarization switching could happen with all the three possible angles (71°, 109° and 180°) when the original OP polarization is antiparallel to the poling field. In addition, in polycrystalline BFO films, we also observed switching (as much as 34% of all switched area) with original OP polarization parallel to the poling field. After analyzing various switching demonstrated in the polycrystalline sample, we found that, although the 71° polarization switching is all in all more favorable than 109° and 180° switching, the possibility of any specific switching is determined by the energy related to the charge migration and IP stress. This work is most valuable for the future applications of BFO in multistate memory devices.

## Theoretical Basis

1. Calculation of piezoelectric surface.

PFM mode is based on the measurement of pizeoelectric displacement. Thus it is necessary to get to know the theoretical pizeoelectric displacement of BFO first.

**Figure 1:**
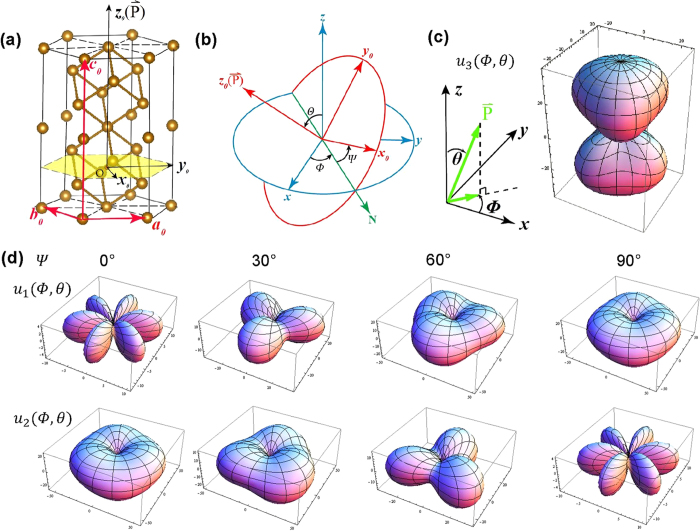
(**a**) Perspective view of the pseudocubic arrangement of Fe ions in the hexagonal representation. (**b**) Schematic for Proper Euler angles. (**c**) Displacement *u*_3_ for BiFeO_3_ on Euler’s angles *Φ*, *θ* in the laboratory coordinate system. (**d**) Displacement *u*_1_ and *u*_2_ for BiFeO_3_ on Euler’s angles *Φ*, *θ* and *Ψ* = 0°, 30°, 60°, 90° in the laboratory coordinate system.

In a crystal coordinate system (Fig. 1a) *x*_*0*_*/y*_*0*_*/z*_*0*_ with the 3-fold axis of BFO along *z*_*0*_ axis 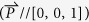 and the basic vectors being 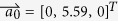

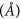
, 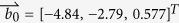

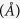
, 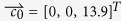

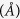
, the piezoelectric tensor is:
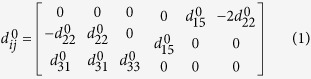
with non-zero coefficients 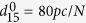, 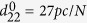, 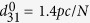 and 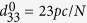 obtained from first-principles calculations (Figure S1).

The grains and unit cells are randomly-oriented in polycrystalline samples. Supposing the crystal coordinate system *x*_*0*_*/y*_*0*_*/z*_*0*_ associates with the laboratory coordinate system *x/y/z* through Euler angles **Φ**, ***θ***, **Ψ** (Fig. 1b), the piezoelectric tensor in the laboratory coordinate system becomes^39^:
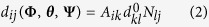
where *A*_*ij*_ is the rotation matrix with Euler angles **Φ**, ***θ***, **Ψ** about axes *z, x, z*.
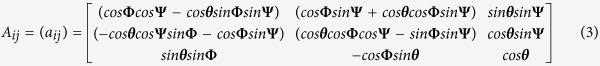

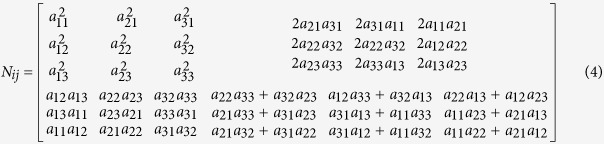


For a domain under a tip voltage of *V*_*Q*_, the piezoelectric displacement along *x, y, z* axes are respectively as follows^43^:
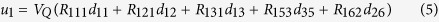

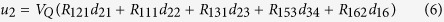

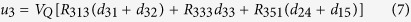
where *R*_111_ = −(13 + 4*v*)/32, *R*_121_ = (1 − 12*v*)/32, *R*_131_ = −1/8, *R*_153_ = −3/8, *R*_162_ = −(7 − 4*v*)/32, *R*_313_ = −(1 + 4*v*)/8, *R*_333_ = −3/4, *R*_351_ = −1/8 and *v* = 0.25.

The calculated piezoelectric face from formulas (2–7) is shown in Fig. 1c,d. It can be clearly seen that *u*_1_, *u*_*2*_, and *u*_*3*_ change with the orientation of BFO grain (corresponding to a set of Euler angles **Φ**, ***θ***, **Ψ**). The sign of *u*_3_ corresponds to that of the polarization component along *z* axis (*P*_*z*_), that is, *u*_3_ > 0 (*u*_3_ < 0) corresponds to *θ* < 90° (*θ* > 90°) and *P*_*z*_ > 0 (*P*_*z*_ < 0).

2. Determination of the switching angles from 2D PFM images.

The switching angles of polarization can be determined through carefully analyzing the PFM signals, and our data processing includes the following four steps:

Step 1: Normalize PFM signals before and after switching.

Our IP_x_ and OP PFM signals, *A*_1_ and *A*_3_, are proportional to the piezoelectric displacements along *x* and *z* axes, respectively, which need to be normalized^39^ by
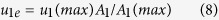

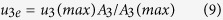
where *A*_1_ and *A*_3_ are the measured IP_x_ and OP PFM signals, respectively, at each point; *A*_1_(*max*) and *A*_3_(*max*) are corresponding maximum values obtained in the two directions within a large scanning area; *u*_1_(*max*) and *u*_3_(*max*) are the maximum values of *u*_1_(**Φ**, ***θ***, **Ψ**) and *u*_3_(**Φ**, ***θ***, **Ψ**) calculated from formula (5) and (7), respectively. In systems with all possible orientations of polarization present, this procedure is equivalent to using an internal standard for the signals^39^.

Using formula (8) and (9), the IP_x_ and OP PFM signals before and after polar scan could be normalized to be (*u*_1*e*_′, *u*_3*e*_′) and (*u*_1*e*_′′, *u*_3*e*_′′), respectively.

Step 2: Obtain theoretical piezoelectric displacements before switching.

Supposing the orientation of the domain before switching is characterized by Euler angles **Φ**’,***θ***’,**Ψ**’, and adopting formulas (2–7), the theoretical displacements along *x* and *z* axes can be given as functions of Euler angles:
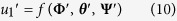

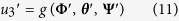


Step 3: Provide all the possible theoretical piezoelectric displacements after switching.

For a BFO domain with a certain direction, there would be all in all seven possible switching cases, since BFO has eight equivalent polarization directions. Suppose the basic vectors of the unit cell (in the laboratory coordinate system) before switching are:
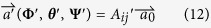

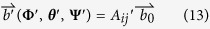

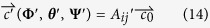


Then after switching, the basic vectors could be
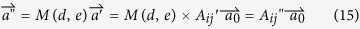

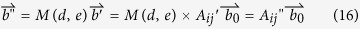

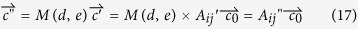
where
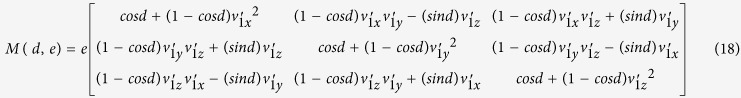
which is the rotation matrix from basic vectors before switching to that after switching, with the permutation of two parameters *d* and *e* among several discrete values corresponding to all the seven possible switching cases, and *v*′_1*x*_, *v*′_1*y*_, *v*′_1*z*_ being the components of the rotation axis (unit vector) along *x*, *y*, *z* axes respectively. (Figure S2).

*A*_*ij*_′′ is the rotation matrix from 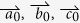 to 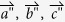. Then resembling formula (2) and in the laboratory coordinate system, the piezoelectric tensor of the domain after switching becomes
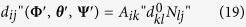


Succeedingly, using formula (5) and (7), the theoretical piezoelectric displacements along *x* and *z* axes after switching can be described as
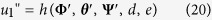

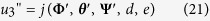


Step 4: Determine the domain orientation and switching angles.

Combining the normalized PFM signals in step 1 and formulas (10–11) and (20–21), we get four equations for each possible switching case:
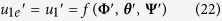

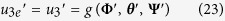

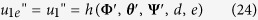

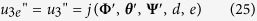
where 0 < **Φ**′ < 2*π*, 0 < ***θ***′ < *π* and 0 < **Ψ**′ < 2*π*. The above four equations could be numerically solved within a certain error range. In the calculation, the step size of (**Φ**′, ***θ***′, **Ψ**′) is set to be (0.01, 0.01, 0.01), and *d* and *e* vary among several discrete values to traverse all the seven possible switching cases as mentioned above. For each domain, the case with the minimal value of the objective function 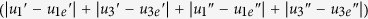 is chosen, so as to determine the orientation and switching angle of the domain (Figure S2).

There are three things worth mentioning. First, although there are underline crystals in polycrystalline films, their impact on the determination of polarization switching on the surface is not critical. The grains with average size of 130 nm in our films are mostly of single-domain state^44^. Assuming a tip radius of 30 nm, the electric field below the 130 nm grain, according to the solution proposed by Mele^45^, would be only 3.7% of that on the surface. Second, in order to eliminate the influence of the irregular grain edges, the average PFM signals over the inner part of a domain was adopted to determine the switching angles. Third, after poling, the same PFM images can be obtained if we repeat the measurement for several times, which confirms the reliability of our signals.

## Results

**Figure 2:**
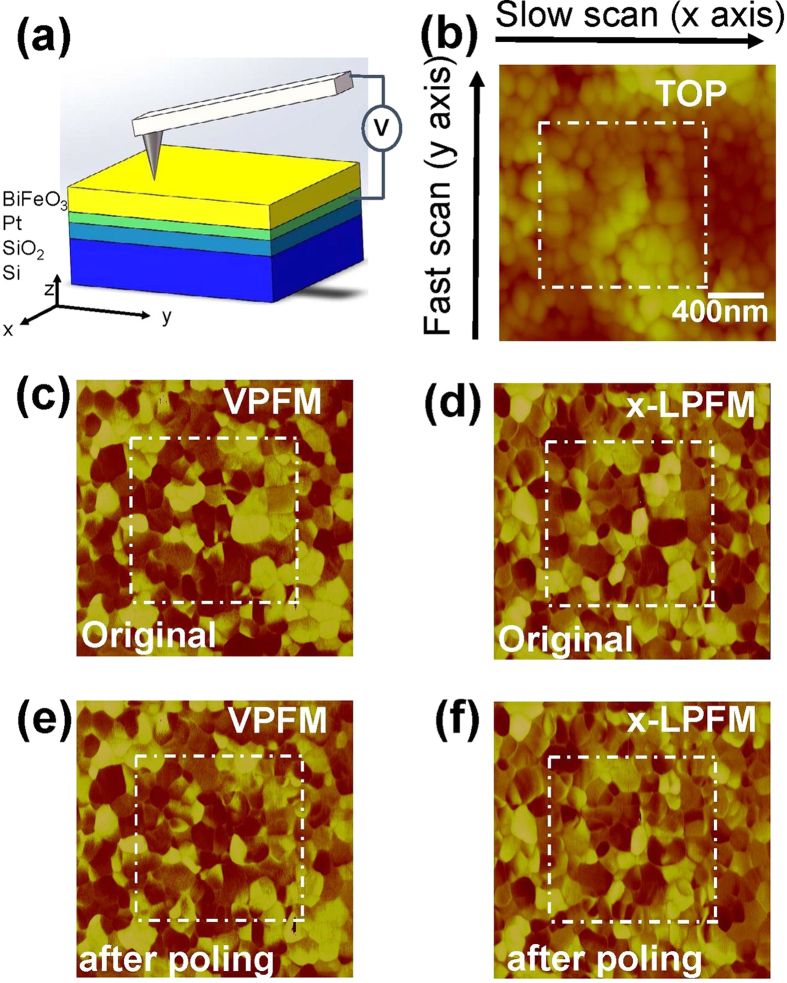
(**a**) Schematic of PFM detection. (**b**) Surface morphology, (**c**) VPFM, (**d**) *x*-LPFM images of the BiFeO_3_ film before and (**e**) VPFM, (f) *x*-LPFM images after poling, where dashed white lines indicate the polar scanned region.

Figure 2b gives the surface morphology of the sample before polar scan, which actually didn’t change after poling. While from the PFM images before (Fig. 2c,d) and after (Fig. 2e,f) polar scan, it can be seen clearly that the piezoresponse signals of the polar scanned region (within dashed white lines in PFM images) dramatically changes after poling.

**Figure 3:**
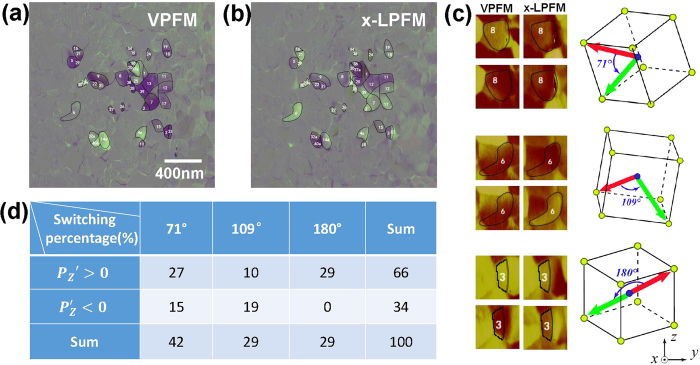
(**a**) Subtracted VPFM [(Fig. 2(e,c,b) *x*-LPFM [(Fig. 2(f,d)] images with 40 switched-domains marked. (**c**) Examples for 71°, 109° and 180° domain switching. (**d**) Statistical data for polarization switching from marked domains.

To clearly present the switched domains, the original PFM signals were subtracted from that after poling, and the processed images are shown in Fig. 3a,b. Purple and white on the grey background indicate switched regions with either VPFM or *x*-LPFM signal change. As mentioned above, the sign of *u*_3_ in Fig. 1c (also sign of VPFM signal) indicates the sign of *P*_*z*_, so purple and white in Fig. 3a represent for normally (Δ*P*_z_ < 0) and abnormally (Δ*P*_z_ > 0) switched regions, respectively. About 85% of the switched area is normally switched, and these domains are numbered without suffix “a” in Fig. 3a,b. The few abnormally switched domains (numbered with suffix “a”) might be related to the relatively rougher surface of polycrystalline samples compared with that of epitaxial films. When the tip moves to a depression, the tip field might induce abnormal polarization switching in nearby higher regions through 71° and 109° switching (no 180° abnormal switching appeared). Although the sign of *u*_1_ in Fig. 1d (also sign of *x*-LPFM signal) has no simple correspondence to the direction of IP_x_ polarization, purple and white area being roughly half-and-half in Fig. 3b still indicates that switching shows no tendentiousness along *x* axis under a poling field along −*z* axis.

Using the data processing method described above, polarization switching with different angles (71°, 109°, and 180°) can be observed in the polycrystalline BFO films (Fig. 3c). All the normally switched domains (abnormally switched domains with rougher surface are not counted in) are assigned into 2 groups, that is, with original OP polarization (OPP) parallel (*θ*′ < 90°, *P*_z_′ > 0) or antiparallel (*θ*′ > 90°, *P*_z_′ < 0) to *z* axis. The statistical data in terms of area are given in Fig. 3d. The understanding of the experimental results bases on the following three points: (a) Electric energy and stress energy are comparable during polarization switching. It is recognized that the polarization switching would happen when the applied electric energy could overcome the corresponding energy barrier, which consists of both electric and stress energy and relates to the switched domain as well as its surroundings. Moreover, our results indicate the electric energy for charge migration and stress energy for lattice distortion are comparable. (b) Electric energy increases with switching angle. Bigger switching angle indicates larger variation of polarization vector, and in turn more energy is needed to fulfil the migration of charge during switching^27^. (c) Stress energy varies for different switching. There is no residual stress for 180° polarization switching since the lattice distortion is the same for the original and the final states; while the 71° and 109° switching are accompanied by additional stress more or less, with the magnitude closely related to the domain orientation before and after switching. Notably, IP stress is vital since OP stress could be easily released on the free film surface.

Now we try to understand the main results in Fig. 3d:

1. Polarization switching could happen no matter the original OPP is antiparallel to the poling field (*P*_z_′ > 0) or not. Among all the switched areas, about 34% (in terms of area) are those with original OPP parallel to the poling field, much larger than that we anticipated. This phenomenon might be related to BFO’s special crystal structure. Even if the original OPP is along the poling field (−*z* axis), there are still switching possibilities (71° or 109°) as long as the component of polarization along –*z* axis is not the maximum among all eight equivalent polarization directions (Figure S3).
2. For 71° switching, about 27% (of all the switched area) are those with original OPP antiparallel to the poling field (*P*_z_′ > 0), larger than that parallel to the field (15%, *P*_z_′ < 0). Although the charge migration are similar for these two cases, the IP stress in the former case is smaller, on average, as qualitatively illustrated in Figure S4a.
3. For 109° switching, about 10% (of all the switched area) are those with original OPP antiparallel to the poling field (*P*_z_′ > 0), smaller than that parallel to the field (19%, *P*_z_′ < 0). Similar to the above-mentioned 71° switching case, the difference comes from the IP stress (Figure S4b) rather than the charge migration.
4. For switching with original OPP antiparallel to the poling field (*P*_z_′ > 0), the percentage of 180° switching (about 29% of all the switched area) is close to that of 71° switching (27%), but obviously higher than that of 109° switching (10%). Although the electric energy related with charge migration for 180° switching is larger than that for 71° switching, 71° switching would be accompanied by IP stress more or less as aforementioned, thus the total energy and in turn the possibilities for these two switching cases could be similar. While either the average IP stress or the migrated charge of 109° switching is greater than that of 71° switching (Figure S4c), the possibility of 109° switching drops significantly.
5. For switching with original OPP parallel to the poling field (*P*_z_′ < 0), no 180° switching appears (as clearly indicated in Figure S3), while the percentage of 71° switching (15% of all the switched area) is a bit smaller than that of 109° switching (19%). The electric energy for charge migration of 71° switching is smaller while the average IP stress is larger than that of 109° switching (Figure S4d), and the latter seems prevail.
6. On the whole, both the percentages of 180° and 109° switching are around 29%, which is distinctly smaller than that of 71° switching (42%). Considering 71° and 109° switching with the original OPP both parallel and antiparallel to the poling field, the stress energy related with these two switching angles are similar, while the electric energy for charge migration of 71° switching is smaller. As to 180° switching, no additional stress energy is related. However, the electric energy related with 180° switching is larger than 71° and 109° switching, and no 180° switching with original OPP parallel to the poling field could occur.

In order to further confirm the above scenario, we scan another area with gradually increasing voltage to 12 V (Figure S5), and find out that the possibilities of various switching under 10 V and 12 V are quite similar to the above observations. Although the statistical data change more or less under lower voltage, the main results are self-consistent. Besides, some new phenomenon arise in supporting our points: Under the lower voltage of 4 V and in the beginning of switching, there is only 71° switching with original OPP antiparallel to the poling field, while other cases of switching happen above 6 V. With the increasing poling voltage up to 10 V, both the percentage of 109° switching and all the switched area increase. As mentioned above, the migrated charge of 71° switching are the minimum among the three switching angles, and the IP stress related with *P*_z_′ > 0 case (Figure S4a) is also small, which makes all in all lowest energy needed for 71° switching with original OPP antiparallel to the poling field. As to 109° switching, the migrated charge is more than that of 71° switching, while the IP stress is larger than that of 180° switching. Thus the 109° switching is the most difficult and the possibility increases with voltage until 10 V when the poling energy is large enough.

Up to now, most of the works concerning switching angles of BFO are about epitaxial films, with which we can compare in the following three aspects: (I) In epitaxial BFO films, the domains usually switches with OPP from antiparallel to parallel to the poling field; while in our polycrystalline films, the domains with original OPP parallel to the field also have a high switching percentage up to 34%. (II) For switching with original OPP antiparallel to the poling field in epitaxial BFO films, it is reported that 109° switching is more difficult and less possible^1^,^24^,^46^, agreeing with our findings. As to 71° and 180° switching, the circumstance is more or less complicated. For (001) epitaxial BFO films, Balke *et al.*^46^ sorted the switching energy by phase-field modeling as: 180° < 71° < 109°, and Zavaliche *et al.*^24^ also indicated that 180° switching is dominant at low fields, while Bake *et al.*^24^ demonstrated a ferroelastic relaxation-mediated 180° switching path through 71° switching. Here in our polycrystalline films, the percentage of 180° and 71° switching with original OPP antiparallel to the poling field are similar (29% and 27%, respectively) after a 12 V polar scan, however, a further study with different poling voltages reveals that 71° switching with *P*_z_′ > 0 happens more easily at lower voltages of 4 V and 6 V. (III) Investigations on epitaxial BFO films indicate that lateral constraints affect greatly and even decisively on the polarization switching behaviour^1^,^47^, here we show that, the impact of IP stress is still comparable with charge migration in polycrystalline BFO films.

## Discussion

In summary, we investigated the polarization switching in polycrystalline BFO films by 2D Piezoresponse Force Microscopy. First, a data processing method is introduced to determine the switching angles in polycrystalline BFO films. Second, BFO polycrystalline sample could demonstrate a variety of switching angles (71°, 109° and 180°) and switching forms (*P*_z_ > 0 → *P*_z_ > 0, *P*_z_ > 0 → *P*_z_ < 0 and *P*_z_ < 0 → *P*_z_ < 0), indicating the possible applications of BFO on multistate memories. Finally, although the overall possibility of 71° switching is obviously larger than that of 109° and 180° switching, the possibility of any specific switching could be greatly affected by two comparable factors, charge migration and IP stress, which in turn are sensitive to the geometric arrangement.

More broadly, our data processing method could be extended to other ferroelectric materials with random orientation. With this method, we can avoid the time-consuming sample-rotating procedure and make it possible to timely investigate the relaxation process of switched domains. This work also provides a framework for exploring an approach about how to control switching mode, so as to achieve remarkable magnetoelectric coupling.

## Methods

### Sample Preparation

Polycrystalline BFO films with thickness of about 300 nm and average grain size of about 130 nm were fabricated on Pt/Ti/SiO_2_/Si substrates via Metal Organic Decomposition method, and their rhombohedral structure was confirmed by X-ray Diffraction patterns^44^,^48^.

### Piezoresponse Force Microscopy and poling

A commercial SPM (Nanoscope IV, DI) connected with two additional lock-in amplifiers (7280 DSP) was used to study the polarization switching. As sketched in Fig. 2a,b, the Pt layer was linked to the sample holder by silver paste to give a uniform bottom electrode of the BFO films, while the conductive tip (Pt coated Si_3_N_4_, DI SCM/PIT7) was contacted to the film surface as the top electrode. A square area (2 μm × 2 μm) of BFO films was first scanned in 2D PFM mode (tip voltage ~2 V AC, 21 kHz; scan velocity ~1 Hz) to simultaneously obtain the vertical *z*-component and lateral *x*-component of piezoresponse (OP and IP_x_ signals, saved as VPFM and *x*-LPFM images, respectively). Then a smaller square area of 1 μm × 1 μm in the center of the previously scanned region was polarized by polar scan (scan velocity ~1 Hz) with a poling voltage of +12 V (i.e. the poling field is along –z axis), which is safe for the tip and sufficiently large for polarization switching. After poling, the original 2 μm × 2 μm region was scanned again in 2D PFM mode. In our experiments, the measured PFM signal is a product of the piezoresponse amplitude and the cosine of the phase^39^. The sequential PFM images were processed afterwards using “ImageJ”, an image-processing program.

## Additional Information

**How to cite this article**: Jin, Y. *et al.* Studying the Polarization Switching in Polycrystalline BiFeO_3_ Films by 2D Piezoresponse Force Microscopy. *Sci. Rep.* **5**, 12237; doi: 10.1038/srep12237 (2015).

## Supplementary Material

Supplementary Information
