# Supplementary material for: Studying the Polarization Switching in Polycrystalline BiFeO3 Films by 2D Piezoresponse Force Microscopy
Source: Sci Rep. 2015 Jul 20;5:12237. doi: 10.1038/srep12237 (PMC4507449; doi:10.1038/srep12237)
Supplement: Supplementary Information [file srep12237-s1.pdf]

Supplementary Information

Studying the Polarization Switching in Polycrystalline $BiFeO_3$ Films by 2D Piezoresponse Force Microscopy

Yaming Jin,[1] Xiaomei Lu,[1,4*] Junting Zhang,[2] Yi Kan,[1] Huifeng Bo,[3] Fengzhen Huang,[1,4] Tingting Xu,[1] Yingchao Du,[1] Shuyu Xiao[1] and Jinsong Zhu[1]

[1]*National Laboratory of Solid State Microstructures and Physics School, Nanjing University, Nanjing 210093, P. R. China*

[2]*Department of Physics, China University of Mining and Technology, Xuzhou 221116, People's Republic of China*

[3]*College of Science, Hebei United University, Tangshan 063009, P. R. China*

[4]*Collaborative Innovation Center of Advanced Microstructures, Nanjing University, Nanjing 210093, P. R. China*

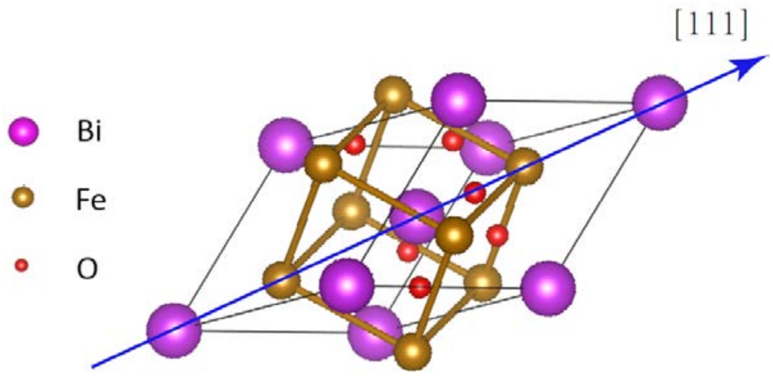

Figure S1. Schematic view of the R3c $BiFeO_3$ (BFO) structure in the rhombohedral representation for first principles calculations.

The piezoelectric tensor of BFO is calculated using the density functional perturbation theory (DFPT), as implemented in the Vienna ab initio simulation package (VASP). The calculations were performed using the projector-augmented wave method within the local spindensity approximation plus the on-site repulsion (LSDA + U), in which Ueff = 4 eV is used on Fe3d states. We use a 5×5×5 Gamma-centered k-point sampling for the calculations, and adopt a plane-wave cutoff of 800 eV and a convergence threshold of 10^{-7} eV to improve the accuracy of piezoelectric tensor. The ionic positions were first fixed to calculate the electronic contribution to the piezoelectric tensor, and then the ions were relaxed to obtain the ionic contribution to the piezoelectric tensor. The total piezoelectric tensor is the sum of the electronic and ionic contributions.

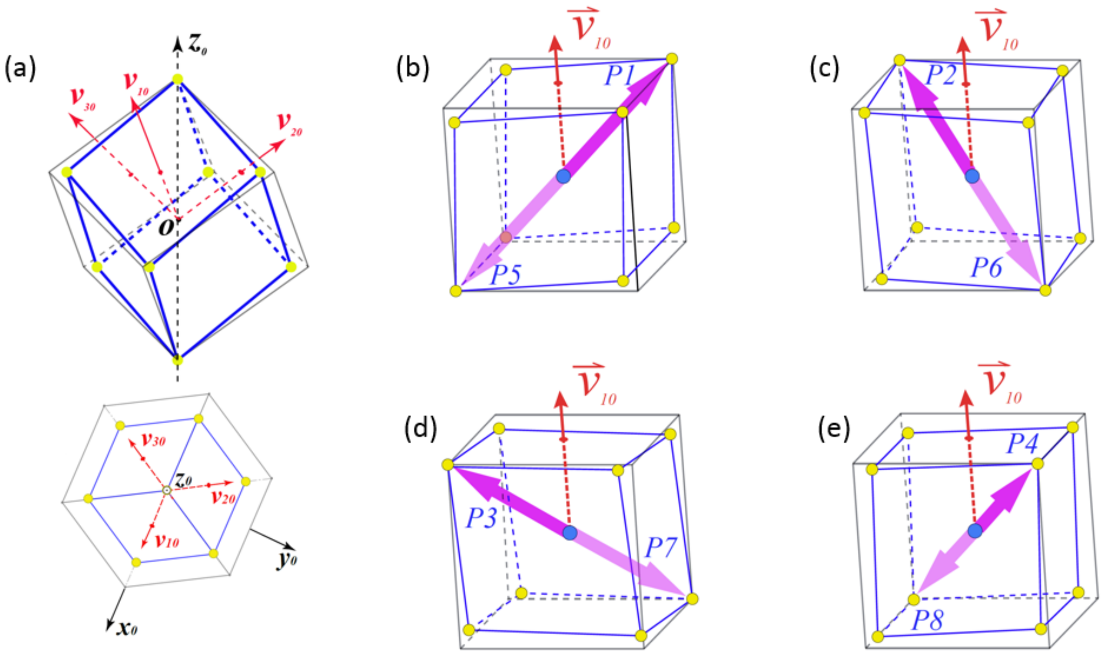

(f)

Parameters	P1	P2	P3	P4	P5	P6	P7	P8
d	0	π/2	π	3π/2	0	π/2	π	3π/2
e	1	1	1	1	-1	-1	-1	-1
switching angle (°)	0	71	109	71	180	109	71	109

Figure S2. Schematic plots for rotation matrixes of all possible switching cases.

The symmetry of BFO at room temperature can be represented by the pseudo cubic arrangement of Bi ions, as depicted by blue lines in Fig. S2(a). This pseudo cube can be just enclosed by a concentric (body center O) cube [grey lines in Fig. S2(a)], the unit basic vectors of which can be presented as $\overrightarrow{v_{10}} = [\sqrt{2}/\sqrt{3}, 0, 1/\sqrt{3}]$, $\overrightarrow{v_{20}} = [-1/\sqrt{6}, 1/\sqrt{2}, 1/\sqrt{3}]$, and $\overrightarrow{v_{30}} = [-1/\sqrt{6}, -1/\sqrt{2}, 1/\sqrt{3}]$ in the crystal coordinate system. Then the exchange among 8 equivalent polarization states could be accomplished by rotating around $\overrightarrow{v_{10}}$, $\overrightarrow{v_{20}}, \overrightarrow{v_{30}}$ and/or inversing in body center. Suppose an original lattice with P1 polarization as depicted in Fig. S2(b), after a rotation of $n\pi/2$ (anticlockwise) around $\overrightarrow{v_{10}}$, the polarization could transform from P1 to P2 [n=1, Fig. S2(c)], P3 [n=2, Fig. S2(d)], and P4 [n=3, Fig. S2(e)]. That is, a rotation of $\pi/2$ around $\overrightarrow{v_{10}}$ corresponds to a 71° polarization switching. After an inversion through the body center, the polarization switches 180° from P2 to P6 [Fig. S2(c)].

In laboratory coordinate system, $\overrightarrow{v_{10}}$ becomes

$$\overrightarrow{v_1}'(\Phi', \theta', \Psi') = A_{ij}{}'\overrightarrow{v_{10}} = (v'_{1x}, v'_{1y}, v'_{1z}). \tag{S1}$$

The transform matrix for 71° polarization switching from P1 to P2 (*i.e.,* an anticlockwise rotation of 90° around $\overrightarrow{v_{10}}$ is

$$M1 = \begin{bmatrix} \cos\frac{\pi}{2} + (1-cos\frac{\pi}{2}){v'_{1x}}^2 & \left(1-cos\frac{\pi}{2}\right)v'_{1x}v'_{1y} - (sin\frac{\pi}{2})v'_{1z} & (1-cosd)v'_{1x}v'_{1z} + (sin\frac{\pi}{2})v'_{1y} \\ \left(1-cos\frac{\pi}{2}\right)v'_{1y}v'_{1z} + (sin\frac{\pi}{2})v'_{1z} & cos\frac{\pi}{2} + (1-cos\frac{\pi}{2}){v'_{1y}}^2 & \left(1-cos\frac{\pi}{2}\right)v'_{1y}v'_{1z} - (sin\frac{\pi}{2})v'_{1x} \\ \left(1-cos\frac{\pi}{2}\right)v'_{1z}v'_{1x} - (sin\frac{\pi}{2})v'_{1y} & \left(1-cos\frac{\pi}{2}\right)v'_{1z}v'_{1y} + (sin\frac{\pi}{2})v'_{1x} & cos\frac{\pi}{2} + (1-cos\frac{\pi}{2}){v'_{1z}}^2 \end{bmatrix}. \quad \text{(S2)}$$

And the matrix for 180° polarization switching from P2 to P6 (*i.e.,* an inversion in the body center) is

$$M2 = \begin{bmatrix} -1 & 0 & 0 \\ 0 & -1 & 0 \\ 0 & 0 & -1 \end{bmatrix}. \quad \text{(S3)}$$

Then a 109°-switching is achieved by the combination of the above two operations from P1 to P6, with the transform matrix being

$$\mathrm{M3} = \mathrm{M1} * \mathrm{M2} = -1\begin{bmatrix} \cos\frac{\pi}{2} + (1-cos\frac{\pi}{2}){v'_{1x}}^2 & \left(1-cos\frac{\pi}{2}\right)v'_{1x}v'_{1y} - (sin\frac{\pi}{2})v'_{1z} & (1-cosd)v'_{1x}v'_{1z} + (sin\frac{\pi}{2})v'_{1y} \\ \left(1-cos\frac{\pi}{2}\right)v'_{1y}v'_{1z} + (sin\frac{\pi}{2})v'_{1z} & cos\frac{\pi}{2} + (1-cos\frac{\pi}{2}){v'_{1y}}^2 & \left(1-cos\frac{\pi}{2}\right)v'_{1y}v'_{1z} - (sin\frac{\pi}{2})v'_{1x} \\ \left(1-cos\frac{\pi}{2}\right)v'_{1z}v'_{1x} - (sin\frac{\pi}{2})v'_{1y} & \left(1-cos\frac{\pi}{2}\right)v'_{1z}v'_{1y} + (sin\frac{\pi}{2})v'_{1x} & cos\frac{\pi}{2} + (1-cos\frac{\pi}{2}){v'_{1z}}^2 \end{bmatrix}. \quad \text{(S4)}$$

A general transform matrix for all the possible switching cases starting from P1 state can be

$$\mathrm{M}(\mathrm{d},\mathrm{e}) = \mathrm{e}\begin{bmatrix} \mathrm{cosd} + (1-cos\mathrm{d}){v'_{1x}}^2 & (1-cos\mathrm{d})v'_{1x}v'_{1y} - (sin\mathrm{d})v'_{1z} & (1-cosd)v'_{1x}v'_{1z} + (sin\mathrm{d})v'_{1y} \\ (1-cos\,\mathrm{d})v'_{1y}v'_{1z} + (sin\mathrm{d})v'_{1z} & cos\mathrm{d} + (1-cos\mathrm{d}){v'_{1y}}^2 & (1-cos\mathrm{d})v'_{1y}v'_{1z} - (sin\mathrm{d})v'_{1x} \\ (1-cos\mathrm{d})v'_{1z}v'_{1x} - (sin\mathrm{d})v'_{1y} & (1-cos\mathrm{d})v'_{1z}v'_{1y} + (sin\mathrm{d})v'_{1x} & cos\mathrm{d} + (1-cos\mathrm{d}){v'_{1z}}^2 \end{bmatrix}, \quad \text{(S5)}$$

where d is the rotation angle (anticlockwise) around $\overrightarrow{v_{10}}$, and e = -1 (e = +1) represents for inversion (identity) operation, with the details listed in Fig. S2(f).

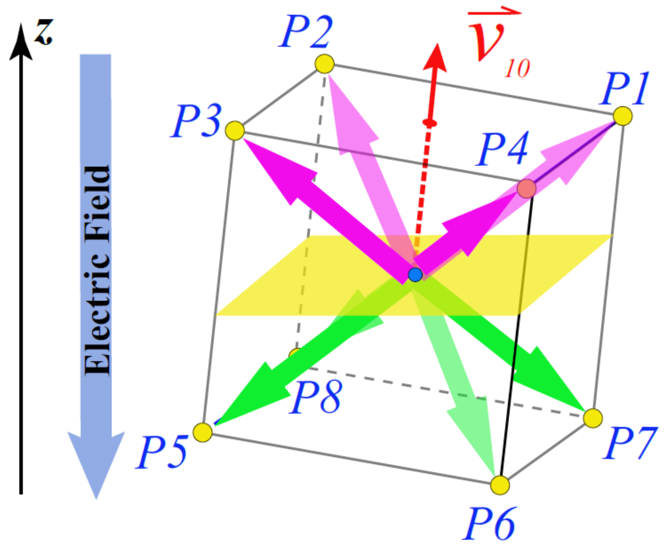

Figure S3. Polarization switching in $BiFeO_3$ under an electric field along –z axis.

The poling field tends to increase the OP polarization along –z axis. Even if the original OP polarization is along –z axis, there are still switching possibilities in BFO. Take crystal lattice as depicted in Figure S3 for instance, the OP polarization of P5-P8 is along the electric field. P8 could switch 71° to P5 and P7, or switch 109° to P6, as long as the OP polarization of P5, P6, and P7 along the field is larger than that of P8. In addition, P5 could switch 71° to P6 while the switching possibility of P6 is much less since the OP polarization of P6 along the field is the maximum among all the 8 polarization states.

Notably, 180°-switching is unlikely if the original OP polarization is along –z axis, otherwise the OP polarization would turn to antiparallel to the poling field, which is of course energetically unfavorable.

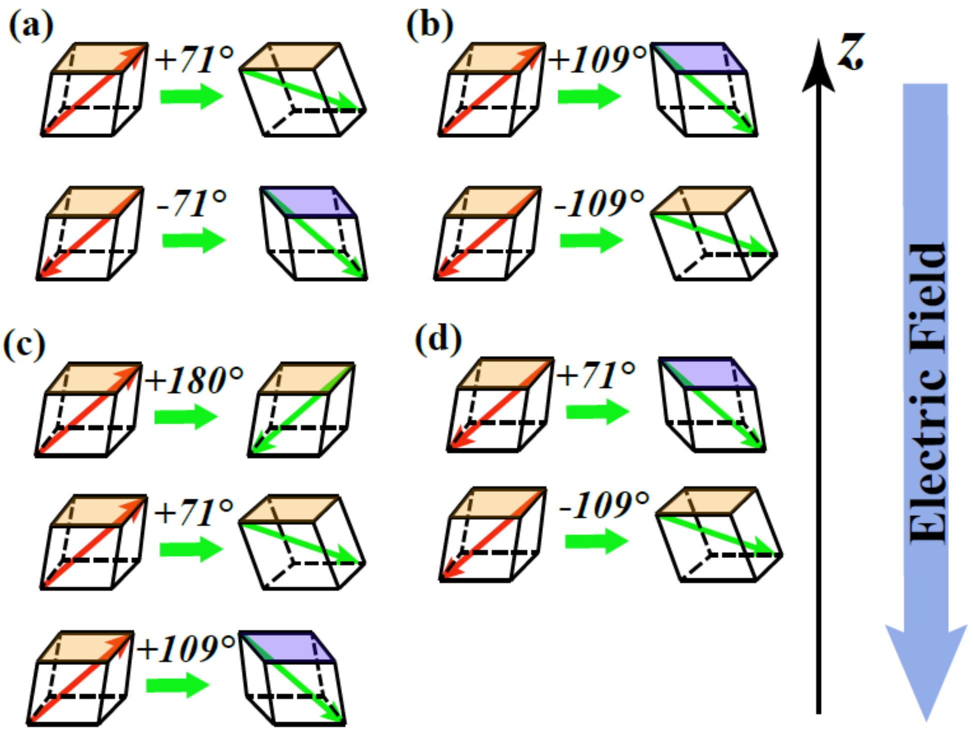

Figure S4. Qualitative illustrations of the in-plane lattice distortion for different switching cases.

The grains and domains are randomly oriented in the polycrystalline sample. In the 2 μm × 2 μm scanning range of our films, the average angle θ between the polarization and the z axis over all $P_Z > 0$ ($P_Z < 0$) regions is about 59.7° (117.6°). To qualitatively compare the IP stress for different switching cases, we suppose a crystal lattice with (001) plane of the Pseudo cubic perpendicular to z axis and the OP polarization after switching along –z direction (poling field). In this state, the angle between polarization and z axis is 54.5° (125.5°) for $P_Z > 0$ ($P_Z < 0$), which is close to the above mentioned average θ. Besides, (001) is a common orientation for epitaxial BFO films [Zhao et al., Nature Mater. **5**, 823; Zavaliche et al., Appl. Phys. Lett. **87**, 252902].

(a) 71° switching with original OP polarization antiparallel (+71°) and parallel (-71°) to the poling field. The latter shows more apparent in-plane distortion.

(b) 109° switching with original OP polarization antiparallel (+109°) and parallel (-109°) to the poling field. The former shows more apparent in-plane distortion.

(c) 71°, 109°, and 180° switching with original OP polarization antiparallel to the poling field. The in-plane lattice distortion of +109° switching is the most apparent, while no additional distortion appears after 180° switching.

(d) 71° and 109° switching with original OP polarization parallel to the poling field. The in-plane lattice distortion of the former is more apparent.

(a)

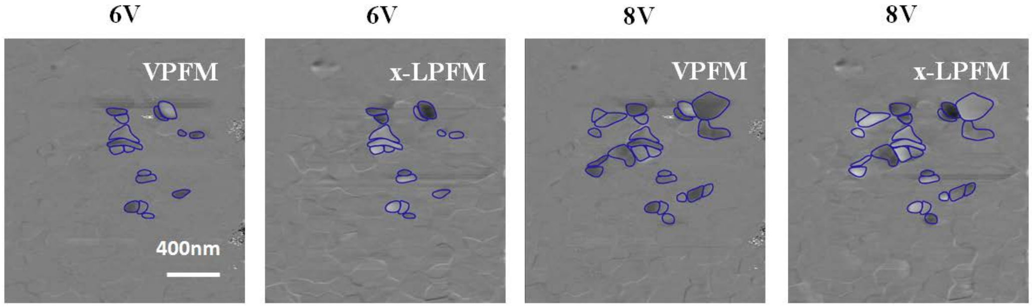

(b)

Switching percentage(%)	71°	109°	180°	Sum
$P'_Z > 0$	28	12	30	70
$P'_Z < 0$	12	18	0	30
Sum	40	30	30	100

(c)

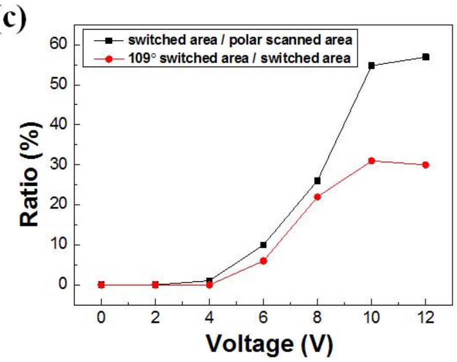

Figure S5. (a) Subtracted VPFM and x-LPFM images with switched-domains for 6 V and 8 V switching voltage, where the original PFM signals were subtracted from that after poling. (b) Statistical data of switched domains after a polar scan of 12 V. (c) Ratio of all the switched area to polar scanned area and the ratio of 109º switched area to all the switched area with increasing poling voltage.
